# CopA Protects *Streptococcus suis* against Copper Toxicity

**DOI:** 10.3390/ijms20122969

**Published:** 2019-06-18

**Authors:** Chengkun Zheng, Mengdie Jia, Tianyu Lu, Miaomiao Gao, Lingzhi Li

**Affiliations:** 1 Joint International Research Laboratory of Agriculture and Agri-Product Safety, The Ministry of Education of China, Yangzhou University, Yangzhou 225009, China; jiamengdie@163.com (M.J.); 18252734942@163.com (T.L.); miaomiaogao1@163.com (M.G.); 18762314176@163.com (L.L.); 2 Jiangsu Key Laboratory of Zoonosis, Yangzhou University, Yangzhou 225009, China; 3 State Key Laboratory of Agricultural Microbiology, Huazhong Agricultural University, Wuhan 430070, China

**Keywords:** CopA, *Streptococcus suis*, copper toxicity, copper resistance

## Abstract

*Streptococcus suis* is a zoonotic pathogen that causes great economic losses to the swine industry and severe threats to public health. A better understanding of its physiology would contribute to the control of its infections. Although copper is an essential micronutrient for life, it is toxic to cells when present in excessive amounts. Herein, we provide evidence that CopA is required for *S. suis* resistance to copper toxicity. Quantitative PCR analysis showed that *copA* expression was specifically induced by copper. Growth curve analyses and spot dilution assays showed that the Δ*copA* mutant was defective in media supplemented with elevated concentrations of copper. Spot dilution assays also revealed that CopA protected *S. suis* against the copper-induced bactericidal effect. Using inductively coupled plasma-optical emission spectroscopy, we demonstrated that the role of CopA in copper resistance was mediated by copper efflux. Collectively, our data indicated that CopA protects *S. suis* against the copper-induced bactericidal effect via copper efflux.

## 1. Introduction

As an important zoonotic pathogen, *Streptococcus suis* not only causes great economic losses to the swine industry worldwide but is also responsible for severe threats to public health. It leads to meningitis, septicemia, pneumonia, endocarditis, and arthritis in pigs, and is associated with meningitis, septicemia, and streptococcal toxic shock-like syndrome in humans [1,2,3]. Of the 29 serotypes (1–19, 21, 23–25, 27–31, and 1/2) proposed on the basis of the pathogen’s capsular polysaccharides, *S. suis* serotype 2 (*S. suis* 2) is generally considered to be the most virulent and the most prevalent in both pigs and humans [4,5,6,7,8,9]. As of 31 December 2013, there have been at least 1642 human cases of *S. suis* infection, with the majority reported in Vietnam, Thailand, and China [10]. In particular, two large outbreaks of human *S. suis* infections in China (in 1998 and 2005, respectively) have changed the opinion that this pathogen only causes sporadic human cases [2,9]. *S. suis* is a persistent threat both to the swine industry and to public health; therefore, a better understanding of the physiology of this agent will undoubtedly contribute to the control of its infections.

Copper, an essential micronutrient for life, functions as a cofactor for a wide variety of enzymes that are involved in various cellular processes [11]. However, an excessive amount of Cu is toxic to cells [11]. Cu has been applied as an antimicrobial agent for thousands of years [12]. Furthermore, the host can utilize Cu toxicity as a mechanism to control bacterial infections [13]. For example, guinea pigs respond to *Mycobacterium tuberculosis* infection by increasing the concentration of Cu in the lung lesions [14]. Moreover, mutation of the Cu-responsive genes results in attenuated virulence in many pathogens [12,13,15]. As a countermeasure, bacteria have evolved several mechanisms to avoid Cu toxicity, including Cu export, Cu sequestration, and Cu(I) oxidation [12]. Among the numerous Cu exporters that have been described, the Cu exporting P_1B_-type ATPases are universally present in bacteria [13]. The most extensively studied Cu-responsive system in Gram-positive bacteria is the *copYZAB* operon of *Enterococcus hirae*, which encodes two P-type ATPases [16]. Similar Cu-responsive operons have been identified in several streptococcal species, such as *Streptococcus mutans* [17,18], *Streptococcus gordonii* [19], *Streptococcus pneumoniae* [20], and *Streptococcus pyogenes* [21]. Nevertheless, no such operon or other Cu-responsive mechanism has been reported in *S. suis*.

In a previous study, we identified two Spx regulators (viz. SpxA1 and SpxA2) in *S. suis*, and found that SpxA1 modulates oxidative stress tolerance and virulence [22]. Although the *copA* gene (encoding a Cu-transporting ATPase) is significantly down-regulated in the Δ*spxA1* mutant, it appears to play no role in oxidative stress tolerance and virulence in *S. suis* [23]. Analysis of the genetic organization of *copA* in *S. suis* revealed that this gene is not arranged in an operon, making it quite distinct from its homologues in certain species of streptococci [17,18,19,20,21]. Thus, we surmised whether CopA could confer protection against Cu toxicity in *S. suis*.

In this study, we examined the role of CopA in Cu tolerance in *S. suis*. Our findings revealed that expression of the *copA* gene was specifically induced in response to Cu. The Δ*copA* mutant exhibited growth inhibition under conditions of excess Cu. Furthermore, we demonstrated that CopA was required for *S. suis* resistance to the Cu-induced bactericidal effect, and the role of CopA in Cu resistance was mediated by Cu efflux.

## 2. Results

### 2.1. S. suis CopA Is a Homologue of the Copper Efflux System

**Figure 1:**
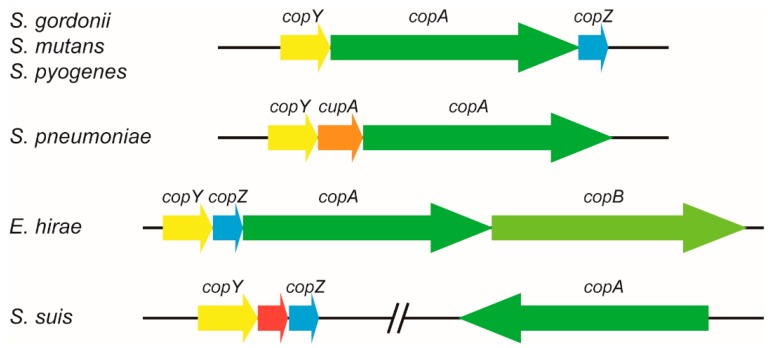
Genetic organization of the *cop* genes in several streptococci and *Enterococcus hirae*. In *Streptococcus suis*, the *copY* and *copZ* genes are separated by a gene (the red arrow) that encodes a hypothetical protein. Arrows indicate the direction of transcription.

**Figure 2:**
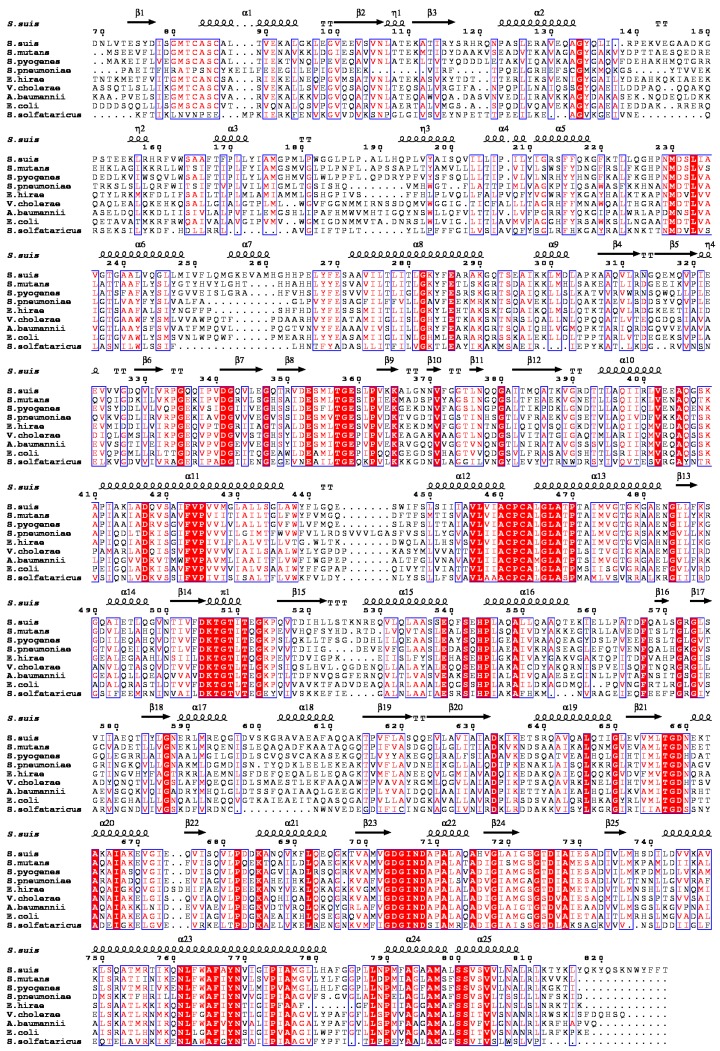
Multiple sequence alignments of CopA homologues. Identical residues are in white letters with red background; similar residues are in red letters with white background. The modelled structure of *Streptococcus suis* CopA is shown on the top. α indicates α-helix; β indicates β-sheet; η indicates coil; and T indicates turn. The GenBank accession numbers are as follows: *S. suis*, WP_012775225.1; *Streptococcus mutans*, NP_720873.1; *Streptococcus pyogenes*, AAZ52023.1; *Streptococcus pneumoniae*, WP_000136284.1; *E. hirae*, WP_131773415.1; *Vibrio cholerae*, NP_231846.1; *Acinetobacter baumannii*, AKA32424.1; *Escherichia coli*, NP_415017.1; and *Sulfolobus solfataricus*, WP_009988559.1.

In *S. suis* 2 strain SC19, CopA encoded by the B9H01_RS06680 locus had 54%, 52%, and 45% amino acid sequence identity to CopA from *S. mutans*, *S. pyogenes*, and *S. pneumoniae*, respectively. In *S. mutans*, *S. gordonii*, and *S. pyogenes*, the genes *copY* (encoding a Cu-responsive transcriptional regulator), *copA*, and *copZ* (encoding a Cu chaperone protein) form a Cu-responsive operon, *copYAZ* (Figure 1) [17,18,19,21]. In *S. pneumoniae*, a *cupA* gene (encoding a hypothetical protein) is present in the operon instead of *copZ* (Figure 1) [20]. The *cop* operon of *E. hirae* consists of four genes that encode CopY, CopZ, CopA, and CopB, respectively (Figure 1) [24]. Unlike the operon organization in these species, the *copA* gene in *S. suis* is far away from the *copY* and *copZ* genes, and these two genes are separated by a gene that encodes a hypothetical protein (Figure 1). Multiple sequence alignment suggested that CopA from prokaryotes shares several conserved motifs (Figure 2). Furthermore, blastn searches revealed that the *copA* gene was present in all complete *S. suis* genomes, with 92% to 100% nucleotide sequence identity (Table 1), indicating that it is highly conserved among a wide range of *S. suis* strains.

### 2.2. S. suis Up-regulates copA Expression in Response to Copper

**Figure 3:**
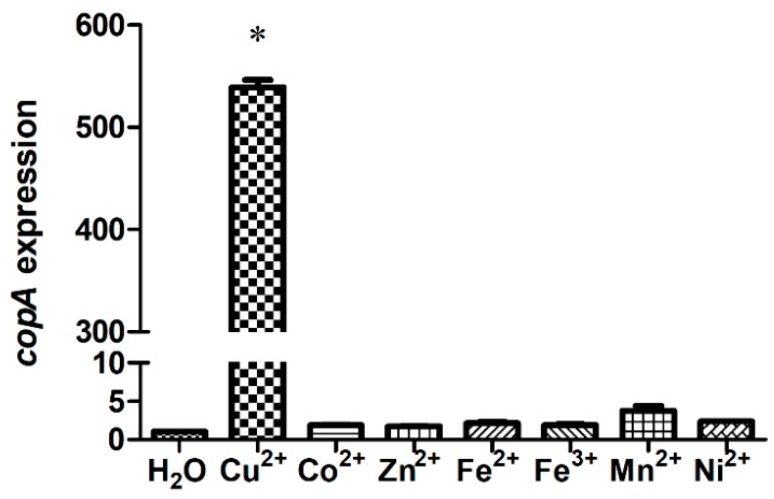
*copA* expression is up-regulated in response to copper. *S. suis* was grown in the presence of various metals, and the gene expression levels were calculated using the 2^−ΔΔ*C*T^ method with 16S rRNA as the reference gene. Results represent the means and standard deviations (SD) from three biological replicates. * indicates *p* < 0.05.

To determine the involvement of *S. suis* CopA in the bacterial resistance to metal toxicity, the *copA* expression levels in the presence of elevated levels of Cu or various other metals were tested. The *copA* expression level of strain SC19 was approximately 530-fold higher in the medium supplemented with 0.5 mM Cu than in the control (Figure 3). In contrast, no significant difference in *copA* expression was detected when the SC19 strain was treated with other metals (Figure 3). Hence, *copA* expression was induced specifically in response to Cu.

### 2.3. CopA Is Required for Copper Resistance in S. suis

**Figure 4:**
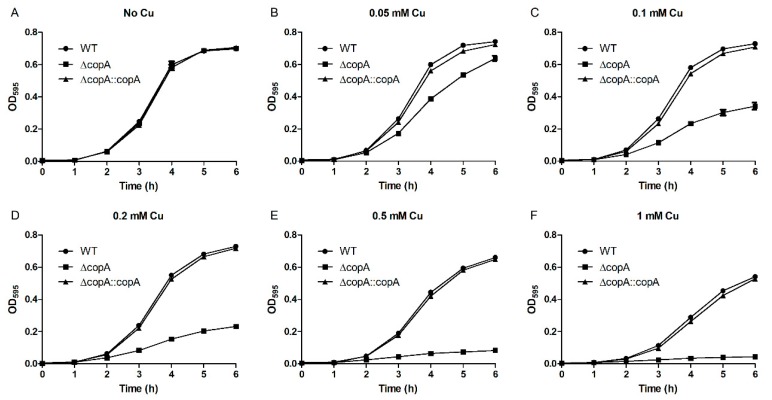
CopA is required for *S. suis* resistance to copper toxicity in liquid medium. Growth curves of the wildtype (WT), Δ*copA*, and Δ*copA*::*copA* strains in the absence (**A**) and presence of 0.05 mM (**B**), 0.1 mM (**C**), 0.2 mM (**D**), 0.5 mM (**E**), and 1 mM (**F**) Cu. The data in the graphs are the means and SD from three wells.

The wild-type (WT), Δ*copA*, and Δ*copA*::*copA* strains were cultured in media supplemented with various concentrations of Cu, and their growth curves were measured to determine the role of CopA in Cu resistance. As seen in Figure 4A, all three strains showed identical growth in the absence of Cu. However, when supplemented with Cu, Δ*copA* clearly exhibited impaired growth compared with the WT and Δ*copA*::*copA* strains (Figure 4B–F). Surprisingly, defective Δ*copA* growth was observed in the presence of as little as 0.05 mM Cu (Figure 4B), and 0.5 mM Cu almost completely inhibited the mutant strain’s growth (Figure 4E).

**Figure 5:**
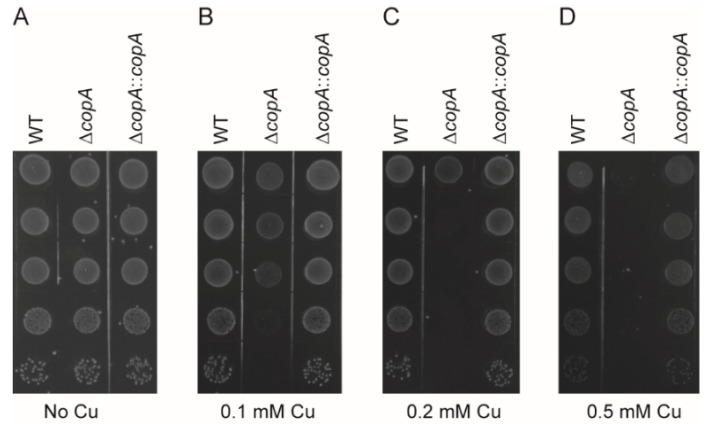
CopA is involved in *S. suis* resistance to copper toxicity in agar plates. Spot dilution assays of the WT, Δ*copA*, and Δ*copA*::*copA* strains in the absence (**A**) and presence of 0.1 mM (**B**), 0.2 mM (**C**), and 0.5 mM (**D**) Cu. Overnight cultures of the strains were serially diluted, and 5 μL of each dilution was spotted onto the plates from 10^−1^ (top) to 10^−5^ (bottom). The graphs are representative of three independent experiments.

The growth defect phenotype of Δ*copA* under Cu excess conditions was also observed on agar plates. The WT, Δ*copA*, and Δ*copA*::*copA* strains all formed colonies with high efficiency in the absence of Cu (Figure 5A). In the presence of Cu, however, Δ*copA* clearly exhibited a decreased ability to form colonies compared with the WT and Δ*copA*::*copA* strains (Figure 5B–D).

**Figure 6:**
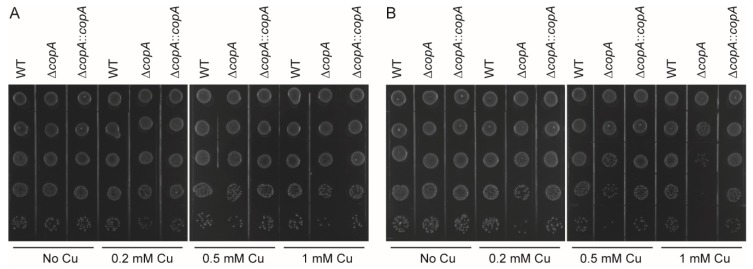
CopA protects *S. suis* against copper-mediated bactericidal effect. The WT, Δ*copA*, and Δ*copA*::*copA* strains were grown to an OD_600_ of 0.6. Each culture was then divided into four equal volumes, which were treated with either varying concentrations of Cu (0.2, 0.5, and 1 mM) or deionized H_2_O. At 2 h (**A**) and 3 h (**B**), aliquots were removed, serially diluted 10-fold up to 10^−5^ dilution, and 5 μL of each dilution was then spotted onto the plates from 10^−1^ (top) to 10^−5^ (bottom). The graphs are representative of three independent experiments.

To determine whether Cu is bactericidal or bacteriostatic and to further assess the role of CopA in Cu resistance, the WT, Δ*copA*, and Δ*copA*::*copA* strains grown to an OD_600_ of 0.6 were treated with H_2_O or various concentrations of Cu, and bacterial survival was analyzed by spot dilution assays. After treatment with Cu for 2 h, Δ*copA* formed a smaller number of colonies than did the WT and Δ*copA*::*copA* strains (Figure 6A). The effect was more prominent after 3 h of treatment (Figure 6B). In contrast, the three strains formed a similar number of colonies following treatment with H_2_O (Figure 6). Thus, Cu is bactericidal to *S. suis*, and CopA protects the bacterium against this effect.

**Figure 7:**
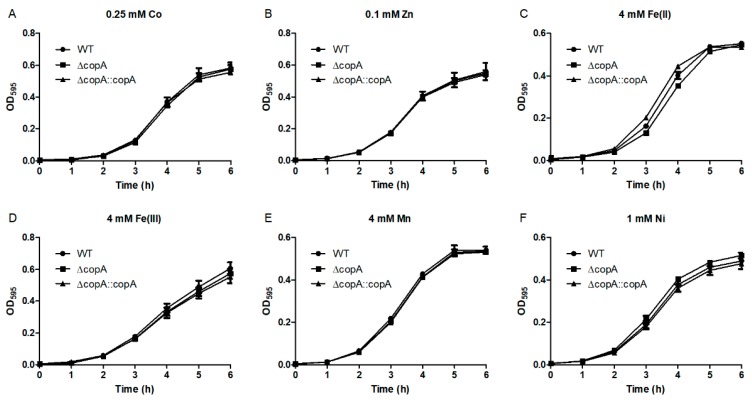
Growth curves of the WT, Δ*copA*, and Δ*copA*::*copA* strains in the presence of various metals. 0.25 mM Co (**A**); 0.1 mM Zn (**B**); 4 mM Fe(II) (**C**); 4 mM Fe(III) (**D**); 4 mM Mn (**E**); 1 mM Ni (**F**).

We also investigated the role of CopA in the bacterial resistance to other metals. As seen in Figure 7, Δ*copA* displayed no growth inhibition effects in the presence of excess Co, Zn, Fe(II), Fe(III), Mn, or Ni. Thus, CopA is specifically required for Cu resistance in *S. suis*.

Taken together, these results indicate that CopA plays an essential role in *S. suis* resistance to the Cu-induced bactericidal effect.

### 2.4. copA Deletion Leads to Increased Intracellular Accumulation of Copper

**Figure 8:**
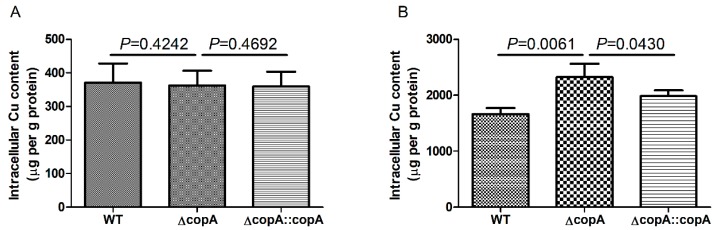
Levels of intracellular copper in the WT, Δ*copA*, and Δ*copA*::*copA* strains. The strains were grown to an OD_600_ of 0.3, and then treated with either H_2_O (**A**) or 0.05 mM Cu (**B**) for 2 h. The intracellular copper content was analyzed by inductively coupled plasma-optical emission spectroscopy (ICP-OES). Results represent the means and SD from three biological replicates.

To understand the mechanism behind the role of CopA in Cu resistance, the intracellular Cu content of the WT, Δ*copA*, and Δ*copA*::*copA* strains grown in the absence or presence of Cu was determined by inductively coupled plasma-optical emission spectroscopy (ICP-OES). When grown in the absence of Cu, the three strains accumulated low and equivalent levels of intracellular Cu (Figure 8A). Following the addition of Cu to the growth medium, a markedly higher level of intracellular Cu was accumulated in all three strains (Figure 8B). However, the intracellular Cu content in Δ*copA* was significantly higher than that in the WT and Δ*copA*::*copA* strains (Figure 8B). These results suggest that the role of CopA in Cu resistance is mediated by Cu efflux.

## 3. Discussion

The present work focused on evaluating the role of CopA in *S. suis* resistance to Cu stress. Our data clearly demonstrated that CopA protects *S. suis* against Cu toxicity, as based on the following lines of evidence: (i) *S. suis* CopA shares a high level of identity (approximately 50%) with its homologues from other streptococcal species, all of which are involved in Cu export [17,18,19,20,21]; (ii) *S. suis* upregulates *copA* expression in response to Cu; (iii) the Δ*copA* mutant exhibits increased sensitivity to Cu stress both in liquid media and on agar plates; (iv) the Δ*copA* mutant forms less colonies after treatment with Cu; and (v) addition of Cu to the medium leads to a higher level of intracellular Cu in the Δ*copA* mutant.

Generally, streptococcal species possess a Cu-responsive operon which participates in Cu resistance [17,18,19,20,21]. Although the genes (i.e. *copY*, *copA*, and *copZ*) that constitute an operon in other species are present in the genome of *S. suis*, they are not arranged into an operon. It has been well established that CopA contributes to Cu resistance in a number of bacteria and archaea, such as *S. pyogenes* [21], *Neisseria gonorrhoeae* [25], *Acinetobacter baumannii* [26], and *Sulfolobus solfataricus* [27]. Likewise, CopA is required for Cu resistance in *S. suis*. In addition, we showed that treatment with Cu leads to the significantly decreased survival of the Δ*copA* mutant, suggesting that Cu is bactericidal to *S. suis*. This claim is consistent with observations in *N. gonorrhoeae* [25] and *M. tuberculosis* [28].

Cu can catalyze the formation of hydroxyl radicals via the Fenton and Haber–Weiss reactions [12,13]. The oxidative damage caused by hydroxyl radical is an important mechanism underlying Cu toxicity [12,13]. Accordingly, the Cu efflux system has been demonstrated to be involved in oxidative stress tolerance in several bacteria [18,26,29]. However, the deletion of *copA* has been shown to have no effect on *S. suis* growth under oxidative stress conditions [23]. *S. suis* possesses multiple regulators and enzymes, such as PerR [30], SpxA1 [22], SrtR [31], superoxide dismutase [32,33], and NADH oxidase [23], to fight against oxidative stress. It is reasonable to speculate that these factors protect Δ*copA* against Cu-induced oxidative stress, resulting in the oxidative stress-tolerant phenotype of this mutant.

The involvement of Cu efflux systems in bacterial pathogenesis has been supported by several lines of evidence. Macrophages use Cu as a defense mechanism against *M. tuberculosis* infection [14]. Furthermore, bacterial virulence is generally attenuated by deletion of the genes that encode the Cu efflux systems [14,20,26]. However, some Cu efflux systems are not required for virulence. For example, several periplasmic proteins are required for Cu tolerance but not for virulence in *Vibrio cholerae* [34]. Similarly, there is no significant difference in survival times between mice inoculated with the WT strain and those inoculated with the Δ*copA* mutant [23]. In line with this finding, a recent study showed that *copA* expression was significantly down-regulated during *S. suis* infection of the blood, joint, and heart of piglets [35].

In conclusion, the evidence provided here clearly demonstrates that CopA is involved in Cu tolerance in *S. suis*. Moreover, the role of CopA in this resistance to Cu-induced bactericidal effect is mediated by Cu efflux.

## 4. Materials and Methods

### 4.1. Bacterial Strains, Plasmids, and Growth Conditions

The bacterial strains and plasmids used in this study are listed in Table 2. The hypervirulent *S. suis* 2 strain SC19 [36] and its isogenic derivatives were routinely grown at 37 °C in Tryptic Soy Broth supplemented with 10% newborn bovine serum (TSBS) or on Tryptic Soy Agar supplemented with 10% newborn bovine serum (TSAS). *Escherichia coli* strain DH5α was cultured at 37 °C in Luria–Bertani (LB) broth or on LB agar. Spectinomycin was added to the growth medium when required at 50 and 100 μg/mL for *E. coli* and *S. suis*, respectively.

### 4.2. Bioinformatic Analysis

Clustal Omega (https://www.ebi.ac.uk/Tools/msa/clustalo/) was used for the sequence alignment of *S. suis* CopA with its homologous proteins. The result was further processed with ESPript 3.0 (http://espript.ibcp.fr/ESPript/ESPript/). Homology modelling of *S. suis* CopA structure was performed with SWISS-MODEL (https://www.swissmodel.expasy.org/). The presence of the *copA* gene in various *S. suis* strains was detected using blastn searches on the NCBI website.

### 4.3. copA Expression Analysis

*S. suis* 2 strain SC19 was first grown in TSBS to an OD_600_ of 0.6. The culture was then divided into eight equal parts, seven of which were supplemented with 0.5 mM CuSO_4_, 0.25 mM CoSO_4_, 0.1 mM ZnSO_4_, 1 mM FeSO_4_, 1 mM Fe(NO_3_)_3_, 1 mM MnSO_4_, or 1 mM NiSO_4_, respectively. Deionized water (H_2_O) was added to the remaining part, which served as the control. These cultures were further incubated for 15 min, following which the bacterial cells were collected for RNA extraction. Total RNA was isolated using the Eastep Super Total RNA Isolation Kit (Promega, Shanghai, China). The RNA integrity was examined by agarose gel electrophoresis, and the RNA concentration was determined using a NanoDrop spectrophotometer. cDNA was generated from 500 ng of RNA using the PrimeScript RT Reagent Kit with gDNA Eraser (TaKaRa, Dalian, China). Quantitative PCR was performed using TB Green Premix Ex Taq II (TaKaRa, Dalian, China) and the primer pair QcopA1/QcopA2 (Table 3) on the StepOnePlus Real-Time PCR System (Applied Biosystems, Waltham, MA, USA). The levels of *copA* expression were calculated using the 2^−ΔΔ*C*T^ method [38], with 16S rRNA as the reference gene. The differences in gene expression were analyzed using one-way analysis of variance with Bonferroni’s post-test.

### 4.4. Construction of the Complementation Strain

The *copA* gene and its flanking regions were amplified from the *S. suis* genome using the primer pair L1/R2 (Table 3). After digestion with the Sal I and EcoR I enzymes, the PCR fragment was cloned into pSET4s [37], yielding the pSET4s::C*copA* plasmid, which was then electroporated into the Δ*copA* mutant [23]. The same procedures used for mutant construction were followed to create the complementation strain (Δ*copA*::*copA*).

### 4.5. Growth Curve Analyses

Growth curve analyses of the WT, Δ*copA*, and Δ*copA*::*copA* strains were performed using various concentrations of CuSO_4_, CoSO_4_, ZnSO_4_, FeSO_4_, Fe(NO_3_)_3_, MnSO_4_, or NiSO_4_. Overnight cultures of the strains were diluted 1:100 in TSBS supplemented with various amounts of the individual metals. In the case of FeSO_4_, trisodium citrate dihydrate was also added to the medium at a concentration of 1 g/L to reduce iron precipitation. The strains were grown at 37 °C in 96-well plates (200 μL/well), and the OD_595_ values were measured hourly using a CMax Plus plate reader (Molecular Devices, San Jose, CA, USA).

### 4.6. Spot Dilution Assays

Overnight cultures of the WT, Δ*copA*, and Δ*copA*::*copA* strains were serially diluted 10-fold up to 10^−5^ dilution, and 5 μL of each dilution was then spotted onto TSAS plates supplemented with varying concentrations of CuSO_4_ (0, 0.1, 0.2, and 0.5 mM). The plates were incubated at 37 °C for 18 h and then photographically documented.

In another assay, overnight cultures of the WT, Δ*copA*, and Δ*copA*::*copA* strains were diluted 1:100 in TSBS and grown to an OD_600_ of 0.6. Each culture was then divided into four equal volumes that were treated with either deionized H_2_O or varying concentrations of CuSO_4_ (0.2, 0.5, and 1 mM). At 2 and 3 h, aliquots of the cultures were serially diluted 10-fold up to 10^−5^ dilution, and 5 μL of each dilution was then spotted onto TSAS plates. The plates were incubated at 37 °C for 18 h and then photographically documented.

### 4.7. Intracellular Copper Content Analysis

The WT, Δ*copA*, and Δ*copA*::*copA* strains were grown in TSBS to an OD_600_ of 0.3. Each culture was then divided into two equal volumes, which were treated with either deionized H_2_O or 0.05 mM CuSO_4_ for 2 h. The cells were harvested and washed three times with phosphate buffered saline (PBS) containing 0.25 M EDTA followed by three times with PBS. The cells were resuspended in 350 μL of PBS, and part of the suspension was used to measure the total protein content with a Bradford Protein Assay Kit (Sangon Biotech, Shanghai, China). The remaining 300 μL of the suspension was centrifuged, following which the cells were resuspended in 66% nitric acid and digested for 48 h at 70 °C. Next, the samples were diluted to 2% nitric acid and analyzed for Cu content by ICP-OES at Yangzhou University. The differences in intracellular Cu content were analyzed using the one-tailed unpaired *t*-test.

## Figures and Tables

**Table 1:** Sequence identity of the *copA* gene in *S. suis*.

*S. suis* Strains	Locus Tag	Gene Sequence Identity (%) ^1^
LSM102	A9494_06425	100
SC19	B9H01_06680	100
SS2-1	BVD85_06510	100
ZY05719	ZY05719_06610	100
A7	SSUA7_1228	100
P1/7	SSU1214	100
BM407	SSUBM407_0575	100
SC84	SSUSC84_1247	100
S735	-	99
GZ1	SSGZ1_1230	99
SS12	SSU12_1279	99
05ZYH33	SSU05_1385	99
98HAH33	SSU98_1400	99
SH0104	-	97
HA0609	CR542_03955	97
90-1330	AN924_03380	97
NSUI060	APQ97_02765	97
NSUI002	AA105_03890	97
05HAS68	HAS68_0686	97
YB51	YB51_2960	97
D9	SSUD9_0599	97
ST3	SSUST3_0597	97
CS100322	CR541_06915	97
T15	T15_0568	97
SC070731	NJAUSS_1288	97
JS14	SSUJS14_1360	97
ST1	SSUST1_0574	96
ISU2812	A7J09_03980	96
SH1510	DP111_07130	96
GZ0565	BFP66_02780	95
DN13	A6M16_02880	95
6407	ID09_03115	95
TL13	TL13_0615	95
CZ130302	CVO91_03355	95
HN105	DF184_07440	95
HN136	CWM22_09360	95
SRD478	A7J08_03040	92
1081	BKM67_07590	93
0061	BKM66_07040	93
D12	SSUD12_0568	92
HA1003	DP112_07660	92
AH681	CWI26_08525	92

^1^ Gene sequence identity is compared with the *copA* gene of SC19 strain.

**Table 2:** Bacterial strains and plasmids used in this study.

Strain or Plasmid	Relevant Characteristics	Source or Reference
Strains		
SC19	Virulent *S. suis* 2 strain isolated from the brain of a dead pig	[36]
Δ*copA*	*copA* deletion mutant of strain SC19	[23]
Δ*copA*::*copA*	Complemented strain of Δ*copA*	This study
DH5α	Cloning host for recombinant vector	TransGen
Plasmids		
pSET4s	Thermosensitive suicide vector; Spc^R 1^	[37]
pSET4s::C*copA*	pSET4s containing *copA* and its flanking regions	This study

^1^ Spc^R^, spectinomycin resistant.

**Table 3:** Primers used in this study.

Primer	Sequence (5’-3’) ^1^	Size (bp)	Target Gene
QcopA1	AGAGGATAGGGATGAGCAAGATAACT	148	an internal region of *copA*
QcopA2	TTTGTCTGGTCAGCAGCATTTACT		
Q16S1	TAGTCCACGCCGTAAACGATG	159	an internal region of 16S rRNA
Q16S2	TAAACCACATGCTCCACCGC		
L1	CCCCGTCGACAATGAGGGCCAAAACGTC	3758	*copA* and its flanking regions
R2	CGCCGAATTCACCATCGACCAGCACTGAG		
In1	TATCACCGAAAGACCACGAC	629	an internal region of *copA*
In2	ATAATGTTTTTGGCGGCAC		
Out1	GAGGACAAAATCAGGGGCT	2769/378	a fragment containing *copA*
Out2	AGGGAACAGGCTGAAAACC		

^1^ The bold sequences are restriction sites.

## Abbreviations

WT	wild-type
ICP-OES	inductively coupled plasma-optical emission spectroscopy
TSBS	Tryptic Soy Broth supplemented with 10% newborn bovine serum
TSAS	Tryptic Soy Agar supplemented with 10% newborn bovine serum
PBS	phosphate buffered saline
